# Sequence analysis of mitochondrial *ND*1 gene can reveal the genetic structure and origin of *Bactrocera dorsalis s.s.*

**DOI:** 10.1186/1471-2148-14-55

**Published:** 2014-03-21

**Authors:** Zhong-Zhen Wu, Hong-Mei Li, Shu-Ying Bin, Jun Ma, Hua-Liang He, Xian-Feng Li, Fei-Liang Gong, Jin-Tian Lin

**Affiliations:** 1 Institute for Management of Invasive Alien Species, 314 Yingdong teaching building, Zhongkai University of Agriculture and Engineering, Guangzhou 510225, PR China; 2 College of Life Sciences, Zhongkai University of Agriculture and Engineering, Zhongkai University of Agriculture and Engineering, Guangzhou 510225, PR China; 3 Guangdong Inspection and Quarantine Technology Center, Guangdong Entry-Exit Inspection and Quarantine Bureau, Tower A, Guojian Building, No.66 Huacheng Avenue, Zhujiang Xincheng, Guangzhou 510225, PR China

**Keywords:** Genetic structure, Origin, *Bactrocera dorsalis s.s*, Mitochondrial *ND*1

## Abstract

**Background:**

The oriental fruit fly, *Bactrocera dorsalis s.s.*, is one of the most important quarantine pests in many countries, including China. Although the oriental fruit fly has been investigated extensively, its origins and genetic structure remain disputed. In this study, the NADH dehydrogenase subunit 1 (*ND*1) gene was used as a genetic marker to examine the genetic diversity, population structure, and gene flow of *B. dorsalis s.s.* throughout its range in China and southeast Asia.

**Results:**

Haplotype networks and phylogenetic analysis indicated two distinguishable lineages of the fly population but provided no strong support for geographical subdivision in *B. philippinensis*. Demographic analysis revealed rapid expansion of *B. dorsalis s.s.* populations in China and Southeast Asia in the recent years. The greatest amount of genetic diversity was observed in Manila, Pattaya, and Bangkok, and asymmetric migration patterns were observed in different parts of China. The data collected here further show that *B. dorsalis s.s.* in Yunnan, Guangdong, and Fujian Provinces, and in Taiwan might have different origins within southeast Asia.

**Conclusions:**

Using the mitochondrial *ND*1 gene, the results of the present study showed *B. dorsalis s.s.* from different parts of China to have different genetic structures and origins. *B. dorsalis s.s.* in China and southeast Asia was found to have experienced rapid expansion in recent years. Data further support the existence of two distinguishable lineages of *B. dorsalis s.s.* in China and indicate genetic diversity and gene flow from multiple origins.

The sequences in this paper have been deposited in GenBank/NCBI under accession numbers KC413034–KC413367.

## Background

The *Bactrocera dorsalis* (Diptera: Tephritidae) is a major horticultural pest. More than 70 species have been identified within this group [1]. Some of the *B. dorsalis* species are highly destructive because of their wide host-ranges, considerable ecological adaptability, pronounced reproductive potential, and dispersal capacity. The *B. dorsalis s.s.*, *B. papayae*, and *B. philippinensis* species have become invasive in many parts of China. Despite previous studies that have described the subtle morphological characteristics distinguishing these three species [1-3], taxonomists still doubt whether *B. dorsalis* s.s., *B. papayae*, and *B. philippinensis* are truly separate species [4,5].

*B. dorsalis s.s.* was first recorded in Taiwan in 1912 and is now widely distributed in most countries in the Asia-Pacific region [6]. Specifically, it has dispersed from China to the northern parts of the Indian subcontinent over the past 90 years [1]. *B. dorsalis s.s.* has been observed multiple times in China during the past hundred years [7-9]. Currently, *B. dorsalis s.s.* is distributed mostly in China’s southern provinces [10].

Mitochondrial DNA analyses have been conducted to study the genetic structure and origin of *B. dorsalis s.s.* populations in China and Southeast Asia [11-15]. However, the results of these studies have been inconsistent, although it is not known whether these inconsistencies should be attributed to differences in sampling strategies and approaches to analysis. According to Schutze et al., *B. dorsalis s.s.*, *B. papayae*, and *B. philippinensis* should be considered a single species [5]. If this is correct, then analysis of populations sampled from both the ranges of *B. philippinensis* and the *B. dorsalis s.s. /B. papayae* transition zone (e.g., Pattaya and Bangkok, in southern Thailand) should provide clues regarding the invasion events in the region [4]. Although mitochondrial DNA markers have been used to examine the changes in the structures of Chinese populations of *B. dorsalis s.s.* over time, the NADH dehydrogenase subunit 1 (*ND*1) markers in *B. dorsalis s.s.* have not yet been used for an evaluation of the distribution area of *B. philippinensis* or the *B. dorsalis s.s./B. papayae* transition zone [15]. In the present investigation, *ND*1 was used as a genetic marker to study the genetic structure and origin of the *B. dorsalis s.s.* population within China and the surrounding areas. Mitochondrial DNA is suitable for analyses of population genetics because of its relatively high rate of genetic evolution [16-20]. The study included samples from the *B. philippinensis* distribution area and *B. dorsalis s.s. /B. papayae* transition zone to (1) determine the actual number of cryptic species and cryptic lineages of *B. dorsalis s.s.* within China; (2) assess the molecular diversity and genetic architecture of the populations; (3) resolve the controversy of the origin and range expansion of *B. dorsalis s.s.* within China.

## Results

### Two distinct lineages in Chinese *B. dorsalis s.s*

**Figure 1:**
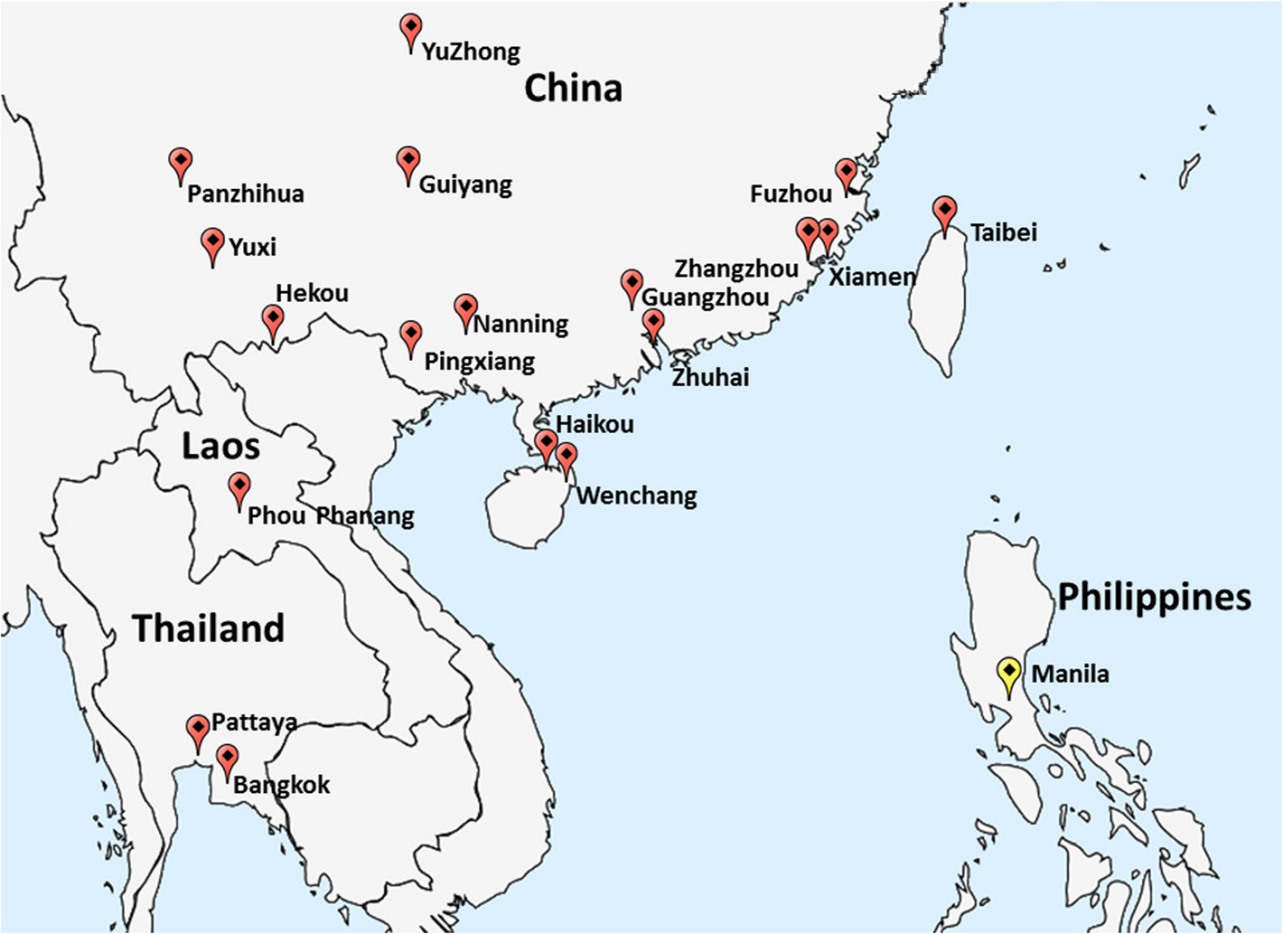
**Collection sites of samples investigated in this study.***B. dorsalis s.s.* is shown in red and *B. philippinensis* is shown in yellow.

**Figure 2:**
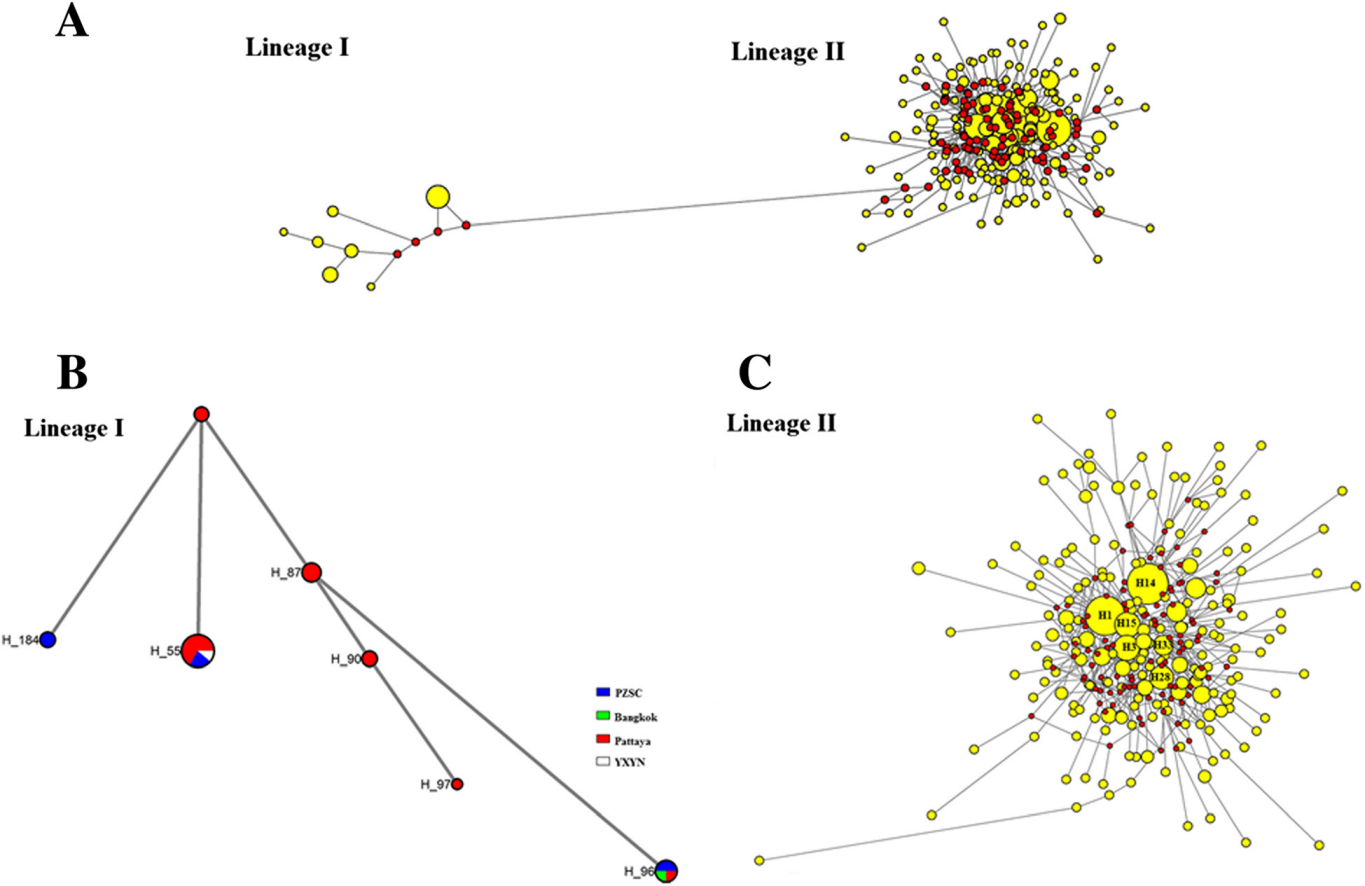
**Median-joining haplotype networks of *ND*1 gene among the samples. A**: The overall networks of haplotypes. The yellow circles represent haplotypes and red dots represent median vector. **B**: Lineage I network. Different sites are shown in different colors. **C**: Lineage II network. The size of the node area is proportional to haplotype frequency.

A total of 19 *B. dorsalis s.s.* populations from China and surrounding areas (Figure 1) were investigated. Analysis of334 *ND*1 sequences from these 19 samples revealed a total of 203 haplotypes, of which 45 were shared and the remaining 158 were single haplotypes. By performing median-joining network analysis, two distinct networks were identified among these 203 haplotypes (Figure 2). The results of the phylogenetic analysis (see Additional file 1: Figure S1) and network analysis clearly showed two distinct lineages, or cryptic species of *B. dorsalis s.s.*, in these samples.

The lineage I network exhibited seven haplotypes (H55, H87, H90, H96, H97, H184, and H186) and the lineage II network showed a star-like pattern with six predominant haplotypes (H1, H3, H14, H15, H28, and H33) (Figure 2). The remaining haplotypes identified from the samples constituted single population groups. The lineage II network mainly included the Pattaya and Panzhihua populations (Figure 2; see Additional file 2: Table S2). The smaller haplotypes of lineage II network, although connected to the central haplotypes, were found frequently (Figure 2; see Additional file 3: Table S2). These results suggest that most haplotypes have a wide geographic distribution throughout China and the surrounding areas. Rare haplotypes were mainly distributed in Pattaya and Panzhihua, showing strict geographic distribution characteristics. These findings suggest that the Pattaya and Panzhihua are mix zones of two distinct lineages, which are here treated as putative species.

### Limited genetic differentiation in geographical populations

To understand the population structure of *B. dorsalis s.s.*, a spatial analysis of molecular variance (SAMOVA) of the *ND1* sequences was performed. The F_CT_ values in the SAMOVA analysis of the lineage II network suggested the existence of three population groups (F_CT_ highest for K = 3). The 19 populations clustered as follows: Guangzhou, Zhuhai, Nanning, Pingxiang, Yuxi, Hekou, Yuzhong, Guiyang, Haikou, Wenchang, Taibei, Bangkok, and Phou Phanang as Subgroup 1; Fuzhou, Zhangzhou, and Xiamen as Subgroup 2; and Manila alone as Subgroup 3. The group comprising most of mainland China, Hainan island, Taiwan island, and southeastern Asia (including mix zones) was found to be the largest subgroup among all the samples (see Additional file 4: Figure S2).

The AMOVA analysis showed inter-subgroup genetic differentiation to account for 12.31% of all differentiation. The genetic differentiation within populations accounted for 86.38%. The analysis also showed genetic differentiation among the three subgroups to be very limited (1.31%) (Table 1). Differentiation among groups (Fct) and within populations (Fst) was not found to be significant. However, differentiation between groups within the same population (Fsc) was (*P* < 0.01) (Table 1). Mantel testing indicated significant association between the pairwise coefficient of genetic differentiation (Fst) and the pairwise geographical distance (*r* = 0.400; *P* = 0.003) within lineage II (see Additional file 2: Table S1). Geographical population cluster levels of genetic differentiation ranged from 0.10207–0.1489 in the Fst values. There was considerable differentiation between the *B. philippinensis* and all other populations (Fst range of 0.10207–0.1489) but moderate amount of differentiation between three other southeast Asian populations (Bangkok, Pattaya, Phou) and all other populations (Fst range of -0.03232–0.20702). The amount of differentiation among *B. dorsalis s.s.* populations within China was also moderate (Fst range of -0.02021–0.10041) (see Additional file 2: Table S1).

**Table 1:** Analysis of molecular variance (AMOVA) for lineage II

**Source of variation**	**df**	**SS**	**VC**	**PV**	**Fixation indices**
Among subgroups	2	41.934	0.40244 Va	12.31	F_SC_ = 0.01491**
Among populations within subgroups	16	56.715	0.04273 Vb	1.31	F_ST_ = 0.13620
Within populations	294	830.102	2.82348 Vc	86.38	F_CT_ = 0.12312
Total	312	928.751	3.26865		

df, degrees of freedom; SS, Sum of squares; VC, Variance components; PV, Percentage of variation; FST, correlation within populations relative to total.

**P < 0.01.

Results obtained from genetic diversity analysis among the 19 geographic populations are summarized in Table 2. The number of haplotypes per population (*Hp*) ranged from 6 to 25. Haplotype diversity (*Hd*) ranged from 0.833 to 1.000, and nucleotide diversity (*π*) ranged from 0.00781 to 0.02586. These differences suggested that all populations retained fairly high levels of genetic diversity. The Manila population showed the most nucleotide diversity (*π*), followed by the Pattaya population.

**Table 2:** Genetic diversity parameters of lineage II

**Populations**	**Code**	**Numbers**	***Hp***	***π*** **± SD**	***k***	***Hd*** **± SD**
China	GZGD	24	18	0.00861 ± 0.00281	4.341	0.957 ± 0.031
	ZHGD	15	13	0.01077 ± 0.00305	5.429	0.971 ± 0.039
	NNGX	13	13	0.01140 ± 0.00313	5.744	1.000 ± 0.030
	PXGX	14	14	0.01180 ± 0.00336	5.945	1.000 ± 0.027
	YXYN	18	13	0.01066 ± 0.00311	5.373	0.928 ± 0.052
	HKYN	18	17	0.00913 ± 0.00294	4.601	0.993 ± 0.021
	YZCQ	12	10	0.01314 ± 0.00329	6.621	0.955 ± 0.057
	PZSC	10	10	0.01230 ± 0.00334	6.200	1.000 ± 0.045
	GYGZ	19	18	0.01305 ± 0.00306	6.579	0.994 ± 0.019
	FZFJ	10	8	0.01067 ± 0.00298	5.378	0.933 ± 0.077
	ZZFJ	13	6	0.00804 ± 0.00212	4.051	0.833 ± 0.081
	XMFJ	20	11	0.00781 ± 0.00231	3.937	0.884 ± 0.054
	HKHN	15	13	0.01270 ± 0.00305	6.400	0.971 ± 0.039
	WCHN	22	20	0.01066 ± 0.00308	5.372	0.991 ± 0.017
	TBTW	31	25	0.00821 ± 0.00267	4.135	0.981 ± 0.015
Thailand	Bangkok	24	22	0.01444 ± 0.00398	7.275	0.993 ± 0.014
	Pattaya	8	7	0.02218 ± 0.00472	11.179	0.964 ± 0.077
Laos	Phou	19	15	0.00953 ± 0.00247	4.801	0.977 ± 0.023
Philippines	Manila	8	8	0.02586 ± 0.00490	13.036	0.964 ± 0.077
Total		313	197	0.01181 ± 0.00379	5.954	0.989 ± 0.002

*π*, Nucleotide diversity, the average number of nucleotide differences per site between two sequences; *k*, the average number of nucleotide differences; *Hd*, Haplotype diversity.

### High migration rates in specific southeast Asian populations

**Figure 3:**
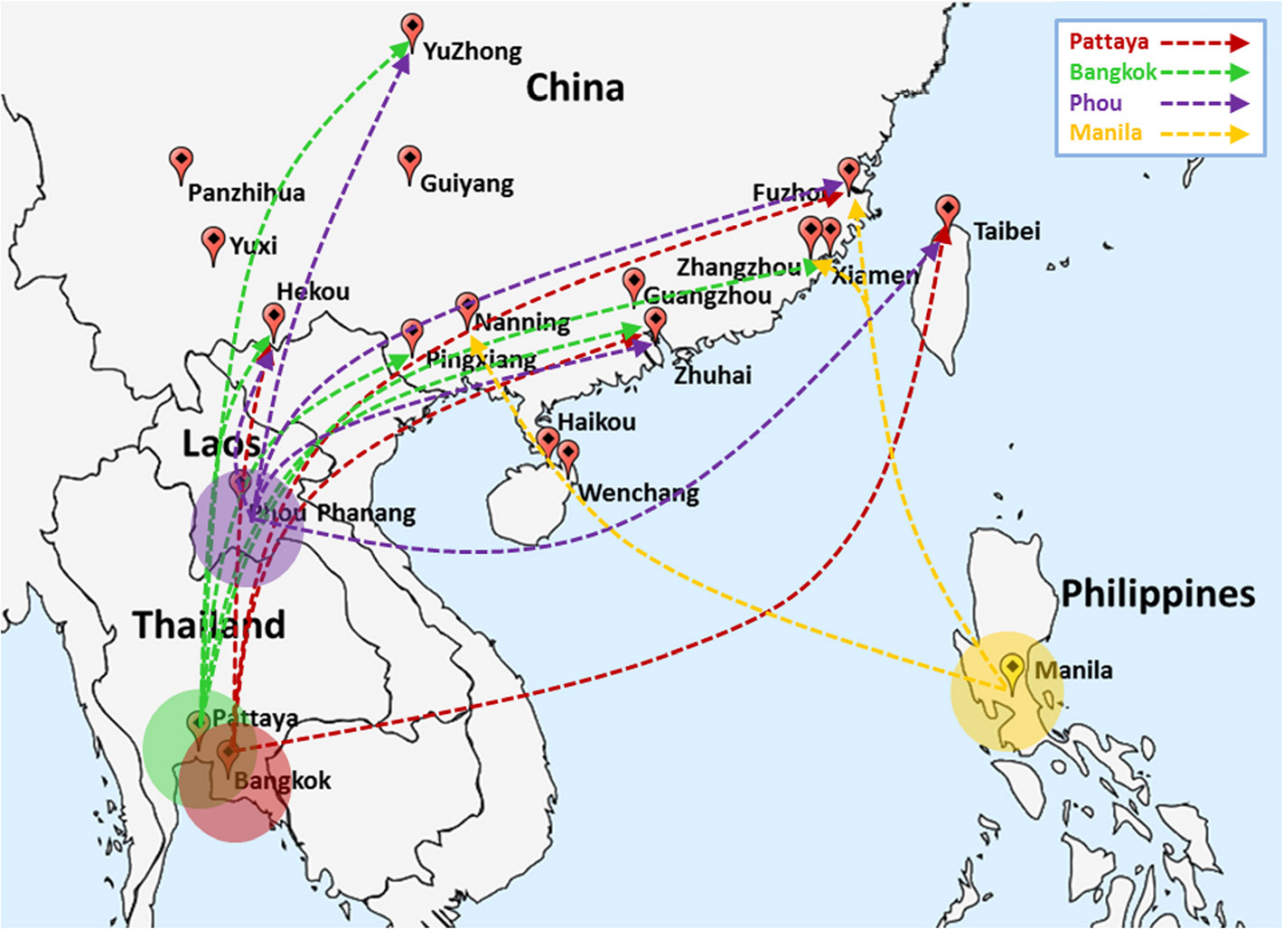
**Direction of migrations between sites.** The predicted origins are shown in four different colors. The direction of migration is also shown.

The migration rate of lineage II was estimated in both directions using MIGRATE. Results are shown in Additional file 5: Table S3. The migration rate ranged from 11.9 (from Pingxiang to Guiyang) to 917.1 (from Hekou to Phou). Specific southeast Asian populations (Bangkok, Pattaya, Phou, and Manila) showed very high migration rates. Asymmetric migration was observed between Manila and Pattaya. A similar situation was observed in populations located from Bangkok to China (Guangzhou, Hekou, Fuzhou, Zhangzhou, and Taibei), from Pattaya to China (Guangzhou, Nanning, Hekou, Yuzhong, Xiamen), from Phou to China (Guangzhou, ZhuHai, Hekou, Yuzhong, Fuzhou, Taibei), and from Manila to China (Nanning, Fuzhou, Zhangzhou). The direction of migration between two sites can be inferred using these asymmetric migration patterns (Figure 3).

### Marked historical population expansion

**Figure 4:**
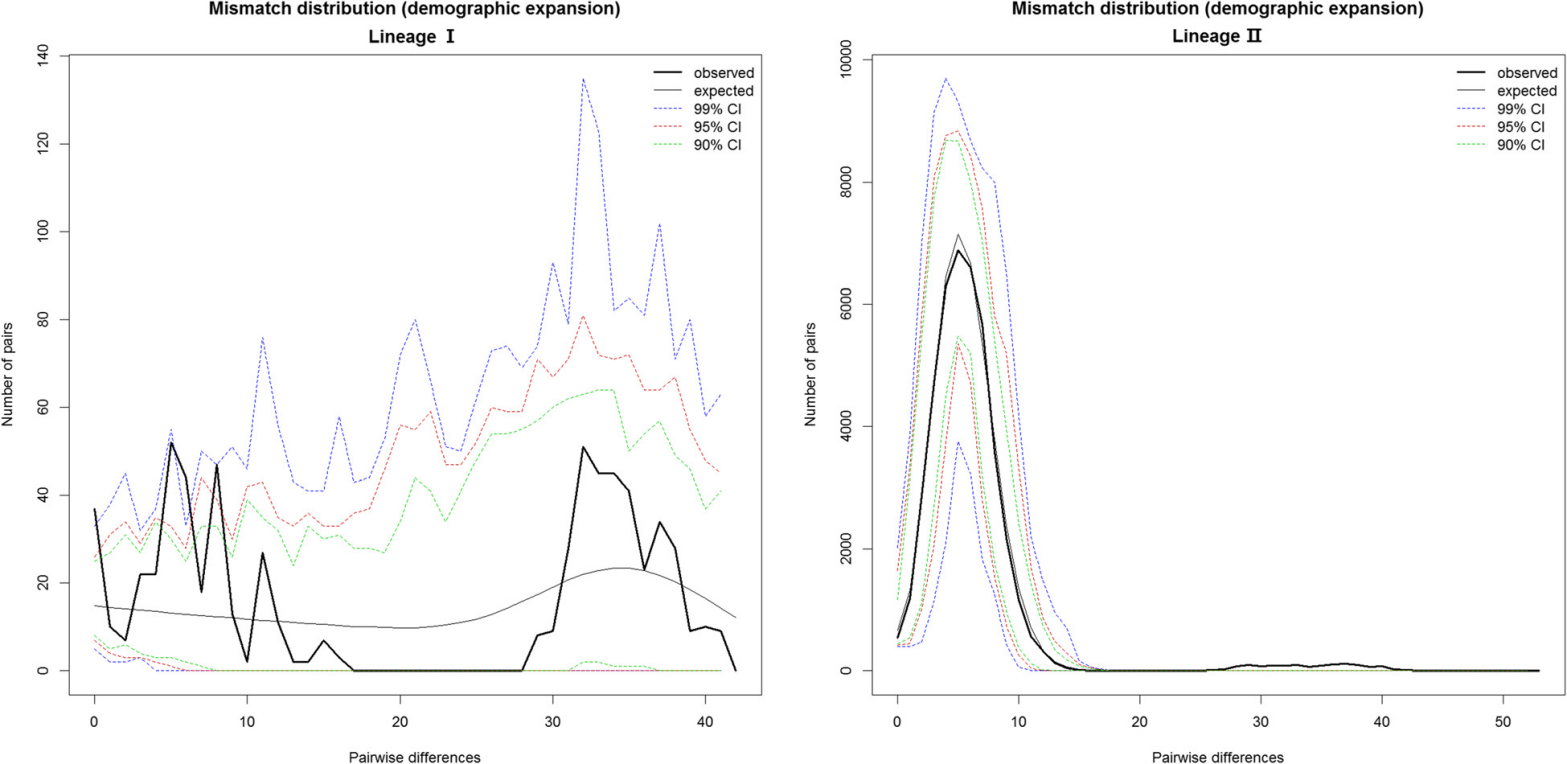
Observed and simulated mismatch distributions of lineage I and lineage II.

The sequence variation of *ND*1 genes was used to perform Tajima’s D test to determine significance of deviations from neutrality. A negative *D* value is produced when more than an expected number of polymorphic sites have low frequencies within a sample. This pattern can be explained by either a recent increase in the size of the population or recent selection [21]. Results indicate negative values (*D* = -1.08524, *P* = 0.17095; Table 3) for lineage II, but these were not found to be statistically significant (*P* > 0.1). This is consistent with a neutral mutation hypothesis for the polymorphisms observed in the *ND*1 gene. Tests of population expansion using Fu’s *Fs* were negative and statistically significant across the entire dataset (*Fs* = -13.36645, *P* = 0.01211). The mismatch distribution of lineage II populations was distinctly unimodal (Figure 4), which is strongly indicative of historical population expansion. In lineage I, both tests of population expansion using Fu’s *Fs* (*Fs* = -24.6437, *P* = 0.93700) and mismatch distribution analysis (Figure 4) suggested that a bottleneck had occurred in lineage I.

**Table 3:** Estimation of sudden population

**Group**	**θ**_**0**_	**θ**_**1**_	**τ**	***D***	***Fs***	***SSD***
Lineage I	0.00176	11.59668	9.32031	-2.2243	-24.6437	0.08150
Lineage II	0.18949	37011.87112	5.70117	-1.08524	-13.36645	0.01940

θ_0_, effective population size before expansion; θ_1_, effective population size after expansion; τ: population expansion time; *D*, Tajiama’s *D*; *Fs*, Fu’s *Fs*; *SSD*, sum of squared deviations between observed and expected mismatch distribution under a sudden expansion model; ^*^P < 0.05; ^**^P < 0.01.

The mismatch distribution of lineage II was compatible with the sudden expansion model (*SSD* = 0.020) (Table 3). The parameters of the expansion model were θ_0_ = 0.190, θ_1_ = 37011.871, and τ = 5.701. The mismatch distribution of lineage I was not compatible with the expansion model (*SSD* = 0.08150). The parameters of the expansion model were θ_0_ = 0.002, θ_1_ = 11.597, and τ = 9.3203 (Table 3). The sudden expansion model was rejected for lineage I (*P* < 0.05). The ratio between estimated effective population size after expansion (θ_0_) and before expansion (θ_1_), which can serve as an estimate of the extent of population growth, was 6589× for lineage I and 195,319× for lineage II.

## Discussion

### Molecular markers and sampling strategy

Molecular markers are useful tools for genetic analysis. They can be used to elucidate the mechanisms underlying biological invasion. They are applicable to the identification of cryptic invasions, reconstruction of invasion history, and assessment of the genetic structure of a population [22]. Mitochondrial genes such as the cytochrome oxidase genes (e.g., *CO*I and *CO*II) have served as molecular makers in studies of the population genetics of invasive insects [23-26]. Although the use of the NADH dehydrogenase gene in such studies is relatively rare, the results of the present study suggest that the NADH dehydrogenase gene is very useful because it shows greater nucleotide substitution rates and more variation than the cytochrome oxidase gene [17-20,27]. Because of this higher rate of variation, the NADH dehydrogenase gene provided a higher resolution in deciphering the geographical population genetics of the recent invasion. NADH dehydrogenase genes have been successfully applied to phylogenetic and population genetic studies of insects, both as an alternative and as a complement to cytochrome oxidase genes [28-30]. Recent investigations of diversity within the *B. dorsalis s.s.* population have focused primarily on mitochondrial genes encoding subunits I or II of cytochrome oxidase (*CO* I or *CO* II) [14,15,31-33]. It is likely that the *B. dorsalis s.s.* found in China is a recent invasion [1,6,7]. NADH dehydrogenase genes could be better suited to the study of recent invasions. Miller et al. suggested that the gene encoding NADH dehydrogenase subunit 1 (*ND*1) may be suitable for the study of geographical populations and genetic evolution in insects [34]. Based on these facts, *ND*1 was chosen for the present study of *B. dorsalis s.s.* population genetics. Earlier claims, in terms of sampling strategy, were not based on an extensive coverage of the distribution of known species. Until recently, *B. dorsalis s.s.*, *B. papayae*, and *B. philippinensis* were thought to belong to different species [2]. However, integrative taxonomy has shown that they are actually a single species [4,5]. For this reason, a larger number of samples were used than in previous studies, and these samples were taken from sites across a broader geographical range (e.g. *B. philippinensis* distribution area and *B. dorsalis s.s./B. papayae* transition zone).

### Genetic structure, demographic history, and identification of cryptic invasions

Evidence from the *ND*1 gene indicated the existence of two distinct lineages that are sympatric within some parts of Pattaya and Panzhihua (Figure 2; see Additional file 1: Figure S1). Of the 334 individuals, 21 were found to belong to the lineage I clade and 313 to lineage II. This disequilibrium can be explained by the existence of two putative species. The first dispersed across a wide geographic area, and the second underwent genetic hitchhiking using external factors (such as infection of maternally inherited microorganisms, such as Wolbachia). The role of Wolbachia in population differentiation and gene flow is known [35]. Previous studies have also found similar phenomena in other species, including Dipteran *Drosophila melanogaster*[36], *Drosophila simulans*[37], *Rhagoletis cerasi*[38], mosquitoes [39], Hemipteran *Bemisia tabaci*[40,41], and Lepidopteran *Danaus chrysippus* (*L*.) [42,43]. These phenomena suggest that infection of maternally inherited microorganisms may influence random mating among specific taxa, leading to divergence [39,42,43]. If Wolbachia had a role in population structure observed in the current study has yet to be examined. In lineage II, a weak genetic structure was observed due to low differentiation among three groups (Fct value was not significant; see Table 1). This is consistent with the results by Wan et al. [15].

Median-joining networks showed that each clade contains both widespread and localized haplotypes (Figure 2). These patterns suggest recent increases in population size and geographical range. Mismatch distribution analysis was conducted to evaluate the history of species expansion [44]. In order to confirm whether or not the *B. dorsalis s.s* population had experienced a large-scale population expansion, the ARLEQUIN 3.5 software package was used to analyze mismatch distribution between the haplotypes’ base variance. The results showed significant differences between observed and expected values of the haplotypes’ base variance along with distinct unimodality distribution patterns of variation (Figure 4). The estimated effective population size after expansion (θ_1_) was 190,000 times higher than before expansion (θ_0_) (Table 3). It was here inferred that the *B. dorsalis s.s* populations of lineage II has experienced rapid expansion in recent years. The average number of nucleotide differences is significantly affected by a population bottleneck, but the number of segregation sites is not [21]. As indicated by the average number of differences in nucleotide sequence (*k*) (Table 2), Chinese *B. dorsalis s.s* populations showed stronger indications of bottleneck and founder events than the southeast Asian populations (Manila, Pattaya, and Bangkok).

Previous taxonomic analyses of the *B. dorsalis* complex were based on geographical subdivisions due to the difficulties of discrimination using morphological traits [2,45]. According to Drew and Hancock, *B. philippinensis* and *B. occipitalis* but not *B. dorsalis s.s* are distributed in the Philippines [2]. However, in the present study, haplotype networks and phylogenetic evidence indicated no strong support for geographical subdivision within the *B. philippinensis* population. In this population, three *B. philippinensis* individuals and the entire *B. dorsalis s.s.* population (Guiyang and Xiamen) were found to belong to the same clade. However, five *B. philippinensis* individuals deviated from the *B. dorsalis s.s.* populations (see Additional file 1: Figure S1). Mantel testing indicated that genetic differentiation was significantly influenced by geographical factors (*r* = 0.400; *P* = 0.003). Geographic distance is probably responsible for this partitioning of genetic variation in the Philippines.

### Invasion history

Nucleotide diversity is the ideal index used to evaluate the degree of variation of nucleotide sites between populations [46]. Dependence can be measured using sample size and sequence length, but genetic diversity can be measured using nucleotide diversity. Nucleotide diversity is the average number of differences in the nucleotide sequence among individuals of a given population, compared pairwise. Generally, ancestors show significantly more genetic diversity than derivative populations because of the founder effect [47,48]. According to this principle, it was here inferred that the populations of *B. dorsalis s.s.* from China might have originated in southeast Asia (Manila, Pattaya, and Bangkok) (Table 2). The MIGRATE analysis showed that gene flow occurred from Manila to Bangkok to Phou to China. However, it occurred in an asymmetric manner. The asymmetric migration of gene flow probably indicates colonization events in China that may have involved multiple sources and sites of invasion. Phylogenetic analysis provided another line of evidence for this: numerous southeast Asian individuals belonged to the same clade as Chinese populations (see Additional file 1: Figure S1). This conclusion is supported by studies showing that southeast Asia might be the region of origin of *B. dorsalis s.s*[11,49]. As in previous studies, the maximum genetic diversity was observed in southeast Asia and Yunnan Province [11,33,49]. However, symmetric migration patterns were observed at a number of sites, distributed mainly in Yunnan, Guangdong, Fujian, and Taiwan. It is likely that these places may have been the first to be invaded. In conclusion, two distinct lineages of *B. dorsalis s.s.* were identified in the present investigation based on sequence data of *ND*1 gene. The asymmetric migration of gene flow indicated multiple invasions and multiple origins.

## Conclusions

Using mitochondrial DNA *ND*1 markers, the genetic structure, origin, and invasion history of *B. dorsalis s.s*. were investigated. It was observed that distinct lineages (both minor and major) originated from specific southeast Asian populations. Interestingly, minor lineages have not spread in China. Evidence was found indicating symmetrical migration from southeast Asia to China. Understanding origin and genetic structure of *B. dorsalis s.s.* will possibly assist in the development of effective management strategies to prevent biological invasion. Source-tracking and minor distinct lineage “encounter” approaches may also provide better clues to the design of appropriate control methods, such as introducing natural enemies, to minimize biological invasion of *B. dorsalis s.s.* in China.

## Methods

### Sample collection, DNA extraction, and sequencing

Adult male flies were collected from 19 natural populations from different locations within China and surrounding areas. All the collections were done during 2007 and 2008. Flies were captured using traps baited with parakairomone methyl eugenol (Table 4; Figure 1). No specific permits were required for these field studies. No specific permission was required to perform these activities in these locations. The locations were not privately owned or protected in any way. The field studies did not involve endangered or protected species. Specimens were preserved in 95% ethanol at -20°C before use. Flies were identified using morphological identification.

**Table 4:** Sample information used in this study

**Location (province)**	**No.**	**GenBank accession no.**	**Code**	**Coordinates (lat./long.)**	**Collect date**
Guangzhou (Guangdong)	24	KC413034-KC413057	GZGD	23.13 N 113.23 E	2008.08
Zhuhai (Guangdong)	15	KC413058-KC413072	ZHGD	22.05 N 113.87 E	2008.08
Nanning (Guangxi)	13	KC413073-KC413085	NNGX	22.82 N 108.37 E	2007.08
Pingxiang (Guangxi)	14	KC413086-KC413099	PXGX	22.12 N 106.73 E	2007.08
Yuxi (Yunnan)	19	KC413100-KC413118	YXYN	24.37 N 102.53 E	2007.08
Hekou (Yunnan)	18	KC413119-KC413136	HKYN	22.58 N 103.33 E	2007.08
YuZhong (Chongqing)	12	KC413137-KC413148	YZCQ	29.58 N 106.55 E	2008.08
Panzhihua (Sichuan)	23	KC413149-KC413171	PZSC	26.58 N 101.72 E	2007.08
Guiyang (Guizhou)	19	KC413172-KC413190	GYGZ	26.58 N 106.70 E	2007.08
Fuzhou (Fujian)	10	KC413190-KC413200	FZFJ	26.13 N 119.05 E	2007.08
Zhangzhou (Fujian)	13	KC413201-KC413213	ZZFJ	24.87 N 117.58 E	2007.08
Xiamen (Fujian)	20	KC413214-KC413233	XMFJ	24.45 N 118.10 E	2007.08
Haikou (Hainan)	15	KC413234-KC413248	HKHN	20.03 N 110.33 E	2008.08
Wenchang (Hainan)	22	KC413249-KC413270	WCHN	19.54 N 110.80 E	2008.08
Taibei (Taiwan)	31	KC413271-KC413301	TBTW	25.05 N 121.50 E	2008.08
Bangkok (Thailand)	25	KC413302-KC413326	Bangkok	13.63 N 101.40 E	2007.08
Pattaya(Thailand)	14	KC413327-KC413340	Pattaya	18.47 N 100.62 E	2007.08
Phou Phanang (Laos)	19	KC413341-KC413359	Phou	18.23 N 102.40 E	2008.08
Manila (Philippines)	8	KC413360-KC413367	Manila	12.87 N 121.77 E	2008.12

Total DNA was extracted from 334 individual specimens using the Universal Genomic DNA Extraction Kit (TaKaRa). The *ND*1 gene fragment of the mitochondrial genome was amplified from all the individuals using the primer *ND*1-F (5′-TTAGTTGCTTGGTTGTGTATTCC-3′) and *ND*1-R (5′-GAAAAAGGTAAAAAACTCTTTCAAGC-3′) [50]. PCR was performed with an initial denaturation step of 95°C for 5 min. The cycle conditions were 95°C for 1 min, 56°C for 1 min, and 72°C for 1.5 min for a total of 35 cycles. PCR ended with a final extension of 72°C for 8 min. PCR products were purified and sequenced using PCR primers from Invitrogen Biotechnology Co. (Shanghai, China). Sequences were deposited in GenBank under accession numbers KC413034–KC413367 (Table 4).

### Data analysis

DNA sequences were assembled using SeqVerter™ and aligned using CLUSTALX 2.0 [51]. They were adjusted manually. Haplotype diversity (*Hd*), nucleotide diversity (*π*), average number of differences in nucleotide sequence between haplotypes (*k*), and standard deviation were determined using DNASP 5.0 [52]. To depict the phylogenetic and geographical relationships of the haplotypic sequences, a haplotype network was created with the median-joining method in NETWORK 4.6 using “connection cost” as the parameter criterion (Frequency > 1 criterion, external rooting, and square option was inactive) [53,54]. A phylogenetic tree was constructed using the neighbor joining method implemented in MEGA 5.0 [55].

The coalescence-based program MIGRATE-N 3.5.1 was used to test for and estimate gene flow between populations [56]. MIGRATE-N estimated past migration rates among populations using a matrix model of asymmetric migration rates. MIGRATE-N was used to estimate the immigration rate M (M = m/*μ*, where m is the immigration rate per generation and *μ* is the mutation rate per generation per locus) among populations. One long chain of 100,000,000 generations was set, with the initial 10,000 excluded as burn-in.

Spatial analysis of molecular variance was performed using SAMOVA 1.0 to identify population groups [57]. Sequence datasets, longitude information, and latitude information served as input data. The greatest number of supported groups (K) was determined by repeating the analysis with K ranging from 2 to 6 and selecting the subdivision scheme associated with highest F_CT_. An AMOVA hierarchical analysis of variance was performed using ARLEQUIN 3.5 to partition total variance into its components among groups, among populations, and within populations [58]. This was based on the identities of the groups inferred by the SAMOVA analysis. Each of the groupings of populations examined using the AMOVA models explained a significant portion of the molecular variance. Genetic differentiation was estimated by calculating Fst between pairs of populations using ARLEQUIN 3.5.

The correlation of genetic differentiation (Fst) over geographic distances for all pairs of populations was tested using the Mantel permutation procedure as implemented in ARLEQUIN 3.5 (1000 permutations, significance level *P* < 0.01) [59].

Demographic history was examined using neutrality statistics of Tajima’s *D* test, Fu’s *Fs* test, and mismatch distribution analysis implemented in ARLEQUIN 3.5 [21,60]. Tajima’s *D* and Fu’s *Fs* tests were carried out to examine the deviations from neutrality expected with population expansion. Significant negative *D* and *Fs* values were considered characteristic of population expansion. Mismatch distribution analysis was performed to evaluate the frequency distribution of pairwise differences between sequences. A unimodal approximate Poisson-like distribution is expected for populations that have experienced demographic expansion in the recent past, but multimodal frequency distribution is expected for populations at equilibrium [61]. Population expansion was further assessed by examining the fitness between the observed and expected frequency distribution with the Harpending’s raggedness index and sum of squared difference (*SSD*) statistics using ARLEQUIN 3.5 [62,63].

## Competing interests

The authors declare that they have no competing interests.

## Authors’ contributions

JTL conceived the ideas for the study; SYB, JM, HLH, XFL, and JTL collected the samples; ZZW and HML performed the experiments; ZZW analyzed the data and composed the manuscript. All authors have read and approved the final manuscript.

## Supplementary Material

Additional file 1: Figure S1 Subgroups of lineage II. The three subgroups are shown by different colors. Click here for file

Additional file 2: Table S1 Haplotypes of *ND*1 identified in each population. Click here for file

Additional file 3: Table S2 Coefficient of genetic differentiation (under diagonal) and geographic distances (above diagonal) of lineage II. Click here for file

Additional file 4: Figure S2 Neighbor-joining tree of lineage I and II. Click here for file

Additional file 5: Table S3 Immigration rate between population pairs estimated using MIGRATE. Click here for file
