# Supplementary figures and images for: Sequence analysis of mitochondrial ND1 gene can reveal the genetic structure and origin of Bactrocera dorsalis s.s
Source: BMC Evol Biol. 2014 Mar 21;14:55. doi: 10.1186/1471-2148-14-55 (PMC3998037; doi:10.1186/1471-2148-14-55)

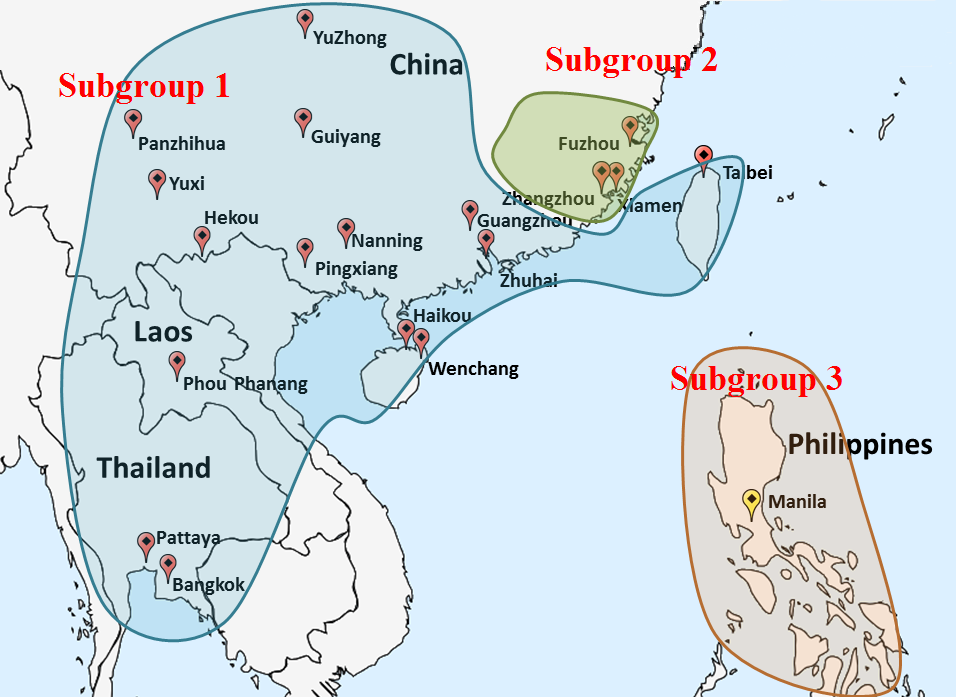

Supplement: Additional file 1: Figure S1 — Subgroups of lineage II. The three subgroups are shown by different colors. [file 1471-2148-14-55-S1.tiff]
