# Supplementary material for: Sequence analysis of mitochondrial ND1 gene can reveal the genetic structure and origin of Bactrocera dorsalis s.s
Source: BMC Evol Biol. 2014 Mar 21;14:55. doi: 10.1186/1471-2148-14-55 (PMC3998037; doi:10.1186/1471-2148-14-55)
Supplement: Additional file 2: Table S1 — Haplotypes of ND1 identified in each population. [file 1471-2148-14-55-S2.doc]

| **Table S1. Haplotypes of ND1 identified in each population** | |
| --- | --- |
| **GZGD** | (H1)5, (H2)1, (H3)1, (H4)2, (H5)1, (H6)1, (H7)1, (H8)2, (H9)1, (H10)1, (H11)1, (H12)1, (H13)1, (H14)1, (H15)1, (H16)1, (H17)1, (H18)1 |
| **ZHGD** | (H1)3, (H2)1, (H10)1, (H19)1, (H20)1, (H21)1, (H22)1, (H23)1, (H24)1, (H25)1, (H26)1, (H27)1, (H28)1 |
| **NNGX** | (H1)1, (H20)1, (H29)1, (H30)1, (H31)1, (H32)1, (H33)1, (H34)1, (H35)1, (H36)1, (H37)1, (H38)1, (H39)1 |
| **PXGX** | (H40)1, (H41)1, (H42)1, (H43)1, (H44)1, (H45)1, (H46)1, (H47)1, (H48)1, (H49)1, (H50)1, (H51)1 |
| **YXYN** | (H52)1, (H53)1, (H54)1, (H55)1, (H56)5, (H57)1, (H58)2, (H59)1, (H60)1, (H61)1, (H62)1, (H63)1, (H64)1, (H65)1 |
| **HKYN** | (H3)1, (H11)1, (H14)3, (H15)1, (H27)1, (H66)1, (H67)1, (H68)1, (H69)1, (H70)1, (H71)1, (H72)1, (H73)1, (H74)1, (H75)1, (H76)1, (H77)2 |
| **YZCQ** | (H78)1, (H79)3, (H80)1, (H81)1, (H82)1, (H83)1, (H84)1, (H85)1, (H86)1 |
| **PZSC** | (H49)1, (H87)3, (H88)1, (H89)1, (H90)2, (H91)1, (H92)1, (H93)1, (H94)1, (H95)1, (H96)1, (H97)1, (H98)1, (H99)1, (H55)6, |
| **GYGZ** | (H14)1, (H100)1, (H101)2, (H102)1, (H103)1, (H104)1, (H105)1, (H106)1, (H107)1, (H108)1, (H109)1, (H110)1, (H111)1, (H112)1, (H113)1, (H114)1 |
| **FZFJ** | (H1)1, (H5)1, (H11)1, (H14)3, (H28)1, (H115)1, (H116)1, (H117)1 |
| **ZZFJ** | (H1)2, (H14)5, (H28)2, (H29)1, (H118)1, (H119)1 |
| **XMFJ** | (H1)4, (H4)1, (H14)6, (H19)1, (H28)1, (H120)1, (H121)2, (H122)1, (H123)1, (H124)1, (H125)1 |
| **HKHN** | (H3)1, (H11)1, (H14)3, (H15)1, (H27)1, (H33)1, (H49)2, (H66)1, (H67)1, (H68)1, (H69)1, (H70)1, (H71)1, (H72)1, (H73)1, (H74)1, (H75)1, (H76)1, (H77)2, (H126)1, (H127)1, (H128)1, (H129)1, (H130)1, (H131)1, (H132)1, (H133)1, (H134)1 |
| **WCHN** | (H15)1, (H27)1, (H33)1, (H34)1, (H66)2, (H135)1, (H136)1, (H137)1, (H138)1, (H139)1, (H140)1, (H141)1, (H142)1, (H143)1, (H144)1, (H145)1, (H146)1, (H147)1, (H148)1 |
| **TBTW** | (H1)2, (H2)1, (H3)4, (H12)1, (H29)1, (H33)1, (H116)1, (H120)1, (H128)1, (H137)1, (H149)2, (H150)1, (H151)2, (H152)1, (H153)1, (H154)1, (H155)1, (H156)1, (H156)1, (H157)1, (H158)1, (H159)1, (H160)1, (H161)1, (H162)1, (H163)1 |
| **Bangkok** | (H15)2, (H96)1, (H122)2, (H164)1, (H165)1, (H166)1, (H167)1, (H168)1, (H169)1, (H170)1, (H171)1, (H172)1, (H173)1, (H174)1, (H175)1, (H176)1, (H177)1, (H178)1, (H179)1, (H180)1, (H181)1, (H182)1, (H183)1 |
| **Pattaya** | (H3)1, (H15)2, (H55)2, (H96)2, (H128)1, (H184)2, (H185)1, (H186)1, (H187)1 |
| **Phou** | (H1)1, (H3)1, (H14)1, (H34)1, (H66)1, (H189)2, (H190)2, (H191)2, (H192)1, (H193)1, (H194)2, (H195)1, (H196)1, (H197)1 |
| **Manila** | (H112)2, (H198)1, (H199)1, (H200)1, (H201)1, (H202)1, (H203)1 |
