# Supplementary material for: Sequence analysis of mitochondrial ND1 gene can reveal the genetic structure and origin of Bactrocera dorsalis s.s
Source: BMC Evol Biol. 2014 Mar 21;14:55. doi: 10.1186/1471-2148-14-55 (PMC3998037; doi:10.1186/1471-2148-14-55)
Supplement: Additional file 3: Table S2 — Coefficient of genetic differentiation (under diagonal) and geographic distances (above diagonal) of lineage II. [file 1471-2148-14-55-S3.doc]

| Table S2. Coefficient of genetic differentiation (under diagonal) and geographic distances (above diagonal) of lineage II | | | | | | | | | | | | | | | | | | | |
| --- | --- | --- | --- | --- | --- | --- | --- | --- | --- | --- | --- | --- | --- | --- | --- | --- | --- | --- | --- |
|  | GZGD | ZHGD | NNGX | PXGX | YXYN | HKYN | YZCQ | PZSC | GYGZ | FZFJ | ZZFJ | XMFJ | HKHN | WCHN | TBTW | Bangkok | Pattaya | Phou | Manila |
| GZGD |  | 100 | 502 | 678 | 1099 | 958 | 973 | 1221 | 773 | 692 | 472 | 513 | 460 | 473 | 871 | 1704 | 1721 | 1257 | 1247 |
| ZHGD | -0.01029 |  | 536 | 704 | 1151 | 1000 | 1069 | 1288 | 855 | 716 | 483 | 570 | 420 | 420 | 870 | 1679 | 1694 | 1252 | 1154 |
| NNGX | 0.03885 | 0.00096 |  | 184 | 617 | 455 | 766 | 783 | 458 | 1164 | 966 | 1088 | 370 | 442 | 1365 | 1303 | 1345 | 804 | 1611 |
| PXGX | -0.02021 | -0.01790 | 0.02226 |  | 497 | 293 | 825 | 704 | 503 | 1348 | 1145 | 1188 | 436 | 506 | 1546 | 1140 | 1184 | 626 | 1716 |
| YXYN | 0.04538^*^ | 0.02210 | -0.00437 | 0.02691 |  | 247 | 700 | 251 | 482 | 1696 | 1530 | 1575 | 933 | 1004 | 1923 | 1193 | 1271 | 675 | 2212 |
| HKYN | 0.05381^*^ | 0.03189 | -0.01748 | 0.04457 | -0.00234 |  | 821 | 493 | 531 | 1606 | 1415 | 1460 | 717 | 785 | 1816 | 1040 | 1104 | 501 | 1999 |
| YZCQ | 0.00988 | -0.01768 | -0.00313 | -0.01044 | 0.01673 | 0.03097 |  | 582 | 321 | 1311 | 1232 | 1273 | 1120 | 1158 | 1562 | 1861 | 1926 | 1322 | 2221 |
| PZSC | 0.00303 | -0.00366 | -0.01129 | -0.00549 | 0.00867 | 0.01854 | -0.01142 |  | 487 | 1725 | 1613 | 1657 | 1133 | 1205 | 1991 | 1420 | 1500 | 903 | 2395 |
| GYGZ | 0.02021 | 0.01004 | 0.00553 | -0.00318 | 0.01805 | 0.03036^*^ | -0.00782 | -0.00638 |  | 1265 | 1131 | 1176 | 825 | 895 | 1505 | 1565 | 1627 | 1019 | 2000 |
| FZFJ | 0.06027^*^ | 0.02938 | 0.01843 | 0.02225 | 0.04618 | 0.04980^*^ | -0.00825 | 0.00886 | 0.03597 |  | 239 | 214 | 1136 | 1131 | 252 | 2393 | 2408 | 1946 | 1282 |
| ZZFJ | 0.02180 | 0.00395 | -0.01734 | 0.00433 | 0.01914 | 0.00829 | -0.00903 | 0.00095 | 0.00961 | 0.01000 |  | 44 | 902 | 895 | 400 | 2161 | 2174 | 1728 | 1151 |
| XMFJ | 0.02107^*^ | 0.01166 | 0.03551^*^ | 0.02647 | 0.03124^*^ | 0.02220 | 0.04360 | 0.02138 | 0.04234^*^ | 0.10041^*^ | 0.02748^*^ |  | 939 | 929 | 357 | 2198 | 2210 | 1769 | 1135 |
| HKHN | 0.02067^*^ | 0.02058 | -0.01819 | 0.02400 | 0.01384 | 0.01391 | -0.00850^*^ | -0.01075 | 0.01440 | 0.04229^*^ | 0.00594 | 0.03614^*^ |  | 72 | 1282 | 1259 | 1272 | 864 | 1281 |
| WCHN | 0.09070^*^ | 0.06039^*^ | 0.01147 | 0.03923^*^ | 0.02823 | 0.02907 | 0.00645 | 0.01346 | 0.02427 | 0.03889 | 0.02880 | 0.06563 | 0.01485 |  | 1266 | 1272 | 1282 | 903 | 1214 |
| TBTW | 0.05167^*^ | 0.02201 | 0.00671 | 0.05178 | 0.01452 | 0.01050 | 0.01838 | 0.01420 | 0.03245 | 0.06285^*^ | 0.01788 | 0.01043 | 0.01680 | 0.01960 |  | 2541 | 2548 | 2124 | 1163 |
| Bangkok | 0.05063^*^ | 0.03496 | 0.12265^*^ | -0.00224 | 0.11076^*^ | 0.16011^*^ | 0.03803 | 0.06931 | 0.08318^*^ | 0.05858 | 0.09042^*^ | 0.12331^*^ | 0.09656^*^ | 0.11205^*^ | 0.15324^*^ |  | 96 | 545 | 2213 |
| Pattaya | 0.08897^*^ | 0.04816 | 0.13333^*^ | 0.04714 | 0.14661^*^ | 0.18688^*^ | 0.08333 | 0.09270^*^ | 0.11135^*^ | 0.08197^*^ | 0.12304^*^ | 0.16042^*^ | 0.12092^*^ | 0.18036^*^ | 0.18382^*^ | -0.03232 |  | 614 | 2176 |
| Phou | 0.08298^*^ | 0.05776 | 0.15141^*^ | 0.03112 | 0.15972^*^ | 0.19548^*^ | 0.08387^*^ | 0.10637^*^ | 0.11559^*^ | 0.09387 | 0.11928^*^ | 0.16089^*^ | 0.13806^*^ | 0.20702^*^ | 0.19661^*^ | -0.02564 | -0.01874 |  | 2036 |
| Manila | 0.23647^*^ | 0.17236^*^ | 0.10207^*^ | 0.15673^*^ | 0.14615^*^ | 0.15398^*^ | 0.10714^*^ | 0.12583^*^ | 0.11719^*^ | 0.13056^*^ | 0.14677^*^ | 0.23238^*^ | 0.10626^*^ | 0.02411 | 0.15983^*^ | 0.21531^*^ | 0.28162^*^ | 0.30379^*^ |  |
| Note: * Represent the difference at the level of significance, P﹤0.05. | | | | | | | | | | | | | | | | | | | |
