# Supplementary material for: Sequence analysis of mitochondrial ND1 gene can reveal the genetic structure and origin of Bactrocera dorsalis s.s
Source: BMC Evol Biol. 2014 Mar 21;14:55. doi: 10.1186/1471-2148-14-55 (PMC3998037; doi:10.1186/1471-2148-14-55)
Supplement: Additional file 5: Table S3 — Immigration rate between population pairs estimated using MIGRATE. [file 1471-2148-14-55-S5.doc]

| Table S3. Immigration rate between population pairs estimated using MIGRATE | | | | | | | | | | | | | | | | | | | |
| --- | --- | --- | --- | --- | --- | --- | --- | --- | --- | --- | --- | --- | --- | --- | --- | --- | --- | --- | --- |
|  | GZGD | ZHGD | NNGX | PXGX | YXYN | HKYN | YZCQ | PZSC | GYGZ | FZFJ | ZZFJ | XMFJ | HKHN | WCHN | TBTW | Bangkok | Pattaya | Phou | Manila |
| GZGD |  | 448.00 | 136.10 | 477.70 | 182.40 | 785.30 | 85.00 | 358.60 | 453.20 | 579.60 | 243.20 | 477.30 | 847.90 | 778.60 | 611.70 | **846.30** | **683.60** | **795.40** | 389.90 |
| ZHGD | 289.90 |  | 304.10 | 56.80 | 398.10 | 329.10 | 705.90 | 709.00 | 516.00 | 234.10 | 177.30 | 464.00 | 106.40 | 382.00 | 131.00 | 325.30 | 102.30 | **715.30** | 203.20 |
| NNGX | 377.00 | 306.00 |  | 549.60 | 617.00 | 578.20 | 221.10 | 865.00 | 650.70 | 623.50 | 677.30 | 129.00 | 796.50 | 261.30 | 807.30 | 291.00 | **827.60** | 271.60 | **789.90** |
| PXGX | 396.60 | 304.00 | 642.30 |  | 251.80 | 101.00 | 131.40 | 484.60 | 359.40 | 609.70 | 28.10 | 300.40 | 453.10 | 238.10 | 240.90 | 206.70 | 278.20 | 153.00 | 35.40 |
| YXYN | 50.30 | 662.80 | 188.80 | 86.80 |  | 246.80 | 304.40 | 850.10 | 780.00 | 582.10 | 768.50 | 243.70 | 704.80 | 866.90 | 153.90 | 139.00 | 244.80 | 450.40 | 181.30 |
| HKYN | 748.20 | 540.30 | 154.40 | 214.20 | 197.40 |  | 166.40 | 757.60 | 424.30 | 370.80 | 629.40 | 322.30 | 851.40 | 552.40 | 693.70 | **530.90** | **631.90** | **589.30** | 417.70 |
| YZCQ | 289.10 | 292.40 | 674.00 | 733.40 | 567.10 | 622.00 |  | 466.40 | 85.30 | 227.90 | 72.90 | 685.50 | 529.20 | 565.40 | 358.60 | 203.90 | **631.40** | **720.50** | 84.90 |
| PZSC | 204.90 | 168.50 | 151.80 | 112.80 | 59.00 | 38.00 | 41.90 |  | 796.60 | 535.10 | 232.10 | 302.90 | 94.20 | 230.00 | 548.40 | 302.80 | 180.30 | 259.70 | 58.50 |
| GYGZ | 87.30 | 202.20 | 48.70 | **11.90** | 133.30 | 336.50 | 341.00 | 163.10 |  | 289.60 | 84.10 | 54.00 | 177.50 | 207.10 | 678.10 | 236.20 | 63.20 | 45.70 | 180.20 |
| FZFJ | 835.60 | 304.10 | 658.90 | 295.00 | 422.20 | 356.80 | 175.20 | 216.00 | 81.00 |  | 773.90 | 915.00 | 669.10 | 498.50 | 398.40 | **894.20** | 450.50 | **535.50** | **759.20** |
| ZZFJ | 167.00 | 745.50 | 397.50 | 506.90 | 87.00 | 287.40 | 545.40 | 399.60 | 137.80 | 753.10 |  | 682.40 | 516.40 | 829.20 | 134.70 | **724.50** | 464.30 | 79.40 | **852.40** |
| XMFJ | 569.50 | 474.10 | 367.00 | 329.20 | 898.40 | 587.60 | 841.30 | 705.90 | 649.90 | 616.70 | 112.10 |  | 161.60 | 867.20 | 791.20 | 323.60 | **816.80** | 440.40 | 347.00 |
| HKHN | 825.40 | 514.60 | 436.70 | 674.20 | 716.50 | 374.70 | 717.90 | 261.40 | 598.30 | 815.70 | 503.90 | 497.40 |  | 536.90 | 737.20 | 205.70 | 158.50 | 186.30 | 98.40 |
| WCHN | 388.30 | 93.80 | 124.80 | 603.00 | 360.90 | 337.50 | 121.50 | 80.90 | 399.10 | 35.30 | 107.60 | 62.40 | 41.50 |  | 44.20 | 272.80 | 185.90 | 258.90 | 341.30 |
| TBTW | 778.20 | 46.20 | 95.50 | 512.80 | 812.00 | 275.70 | 425.90 | 219.90 | 424.00 | 302.10 | 205.00 | 377.70 | 833.80 | 130.80 |  | **859.80** | 358.90 | **841.40** | 383.60 |
| Bangkok | 777.20 | 914.30 | 361.90 | 474.60 | 107.10 | 795.00 | 606.30 | 366.10 | 469.30 | 601.20 | 498.50 | 857.80 | 745.20 | 249.10 | 858.20 |  | **580.30** | **817.60** | **800.20** |
| Pattaya | 724.80 | 696.30 | 629.50 | 812.90 | 905.70 | 450.50 | 604.50 | 683.60 | 582.30 | 609.20 | 333.60 | 863.00 | 354.50 | 656.20 | 742.20 | **872.50** |  | **681.30** | **493.20** |
| Phou | 622.00 | 208.20 | 620.50 | 693.30 | 739.50 | **917.10** | 296.30 | 771.70 | 193.50 | 338.80 | 377.10 | 286.50 | 548.40 | 511.80 | 900.20 | **843.40** | **685.00** |  | **792.00** |
| Manila | 453.60 | 673.50 | 612.60 | 766.20 | 178.20 | 879.70 | 806.60 | 770.20 | 451.60 | 706.00 | 788.10 | 322.60 | 221.50 | 580.60 | 588.80 | **599.90** | 135.50 | **703.80** |  |
